# Lost and Found: The Family of NF-κB Inhibitors Is Larger than Assumed in Salmonid Fish

**DOI:** 10.3390/ijms241210229

**Published:** 2023-06-16

**Authors:** Doret R. van Muilekom, Bertrand Collet, Henrike Rebl, Kristina Zlatina, Fabio Sarais, Tom Goldammer, Alexander Rebl

**Affiliations:** 1 Institute of Genome Biology, Research Institute for Farm Animal Biology (FBN), 18196 Dummerstorf, Germany; muilekom@fbn-dummerstorf.de (D.R.v.M.); sarais@fbn-dummerstorf.de (F.S.); 2 VIM, UVSQ, INRAE, Université Paris-Saclay, 78350 Jouy-en-Josas, France; bertrand.collet@inrae.fr; 3 Department of Cell Biology, Rostock University Medical Center, 18057 Rostock, Germany; henrike.rebl@med.uni-rostock.de; 4 Institute of Reproductive Biology, Research Institute for Farm Animal Biology (FBN), 18196 Dummerstorf, Germany; zlatina@fbn-dummerstorf.de; 5 Faculty of Agriculture and Environmental Sciences, University of Rostock, 18059 Rostock, Germany

**Keywords:** IκB, innate immunity, immune regulation, NF-κB, nfkbia, nfkbie

## Abstract

NF-κB signalling is largely controlled by the family of ‘inhibitors of NF-κB’ (IκB). The relevant databases indicate that the genome of rainbow trout contains multiple gene copies coding for iκbα (*nfkbia*), iκbε (*nfkbie*), iκbδ (*nkfbid*), iκbζ (*nfkbiz*), and *bcl3*, but it lacks iκbβ (*nfkbib*) and iκbη (*ankrd42*). Strikingly, three *nfkbia* paralogs are apparently present in salmonid fish, two of which share a high sequence identity, while the third putative *nfkbia* gene is significantly less like its two paralogs. This particular *nfkbia* gene product, iκbα, clusters with the human IκBβ in a phylogenetic analysis, while the other two iκbα proteins from trout associate with their human IκBα counterpart. The transcript concentrations were significantly higher for the structurally more closely related *nfkbia* paralogs than for the structurally less similar paralog, suggesting that iκbβ probably has not been lost from the salmonid genomes but has been incorrectly designated as iκbα. In the present study, two gene variants coding for iκbα (*nfkbia*) and iκbε (*nfkbie*) were prominently expressed in the immune tissues and, particularly, in a cell fraction enriched with granulocytes, monocytes/macrophages, and dendritic cells from the head kidney of rainbow trout. Stimulation of salmonid CHSE-214 cells with zymosan significantly upregulated the iκbα-encoding gene while elevating the copy numbers of the inflammatory markers interleukin-1-beta and interleukin-8. Overexpression of iκbα and iκbε in CHSE-214 cells dose-dependently quenched both the basal and stimulated activity of an NF-κB promoter suggesting their involvement in immune-regulatory processes. This study provides the first functional data on iκbε—versus the well-researched iκbα factor—in a non-mammalian model species.

## 1. Introduction

The family of NF-κB (nuclear factor kappa-light chain-enhancer of activated B cells)/Rel transcription factors is activated by a broad range of environmental and endogenous cues, including viral and bacterial pathogen-associated molecular patterns (PAMPs) and cytokines [1,2]. The activated NF-κB pathways strongly drive immune and stress responses [3,4], as they direct inflammatory processes and cell growth, differentiation, and survival [5,6,7,8,9].

NF-κB activity is primarily controlled by a dynamic interplay between the inhibitors of NF-κB (IκB) and their opponents, IκB kinases (IKK) [10,11], thereby enabling adaptation to the prevailing circumstances and preventing excessive immune responses [12]. The family of NF-κB inhibitors comprises nine members in mammals [10,13,14,15,16,17,18,19,20,21,22], each with different or mutual affinities for various combinations of NF-κB/Rel dimers [23,24,25]. Similarly, the IκB family is subdivided according to the structural and functional properties of its members. IκBα, IκBβ, and IκBε are considered canonical IκB proteins, as they retain NF-κB/Rel factors in the cytosol by masking their nuclear translocation signals (NLS) [10,15,26,27] or by capturing free NF-κB/Rel factors in the nucleus and exporting them back to the cytoplasm [28]. The atypical IκB proteins IκBζ, IκBδ (aka IκBNS), IκBη, and BCL3 control the transcriptional activity of NF-κB in the nucleus [18,19,25,29]. IκBδ is a repressive regulator [19,30,31], whereas IκBη is an activating regulator [20], and IκBζ and BCL3 act as both repressors and activators of NF-κB-driven gene transcription [25,32,33]. The NF-κB precursor proteins p105/NF-κB1 and p100/NF-κB2 also share sequence similarities with Iκb factors, thereby allowing them to bind and retain preformed NF-κB/Rel proteins in the cytoplasm [14,21].

The ankyrin-repeat motif is the evolutionarily best conserved feature of all IκB proteins [34]. The number of ankyrin repeats determines the binding specificity of IκBs to the Rel homology domain (RHD) of NF-κB/Rel proteins [23,26,34,35,36,37,38], including p105/NF-κB1 and p100/NF-κB2. Mammalian IκB proteins contain 5 to 7 ankyrin repeats about 30 to 33 amino acids in length [39]. In addition to the ankyrin-repeat motifs, the mammalian IκB proteins IκBα and IκBβ contain a C-terminal PEST sequence [40,41], rich in proline (P), glutamate (E), serine (S), and threonine (T) residues. These PEST sequences prevent the NF-κB factors from binding to their response elements, thereby regulating the basal turnover of IκBα and IκBβ [23,40,42,43]. Moreover, all mammalian canonical IκB proteins possess signal-responsive serine residues at their N-termini [27,44,45] that are phosphorylated by the ternary IKK complex (IKKα, IKKβ, and NEMO/IKKγ) following an appropriate stimulus [11]. This phosphorylation triggers the polyubiquitination and proteasomal degradation of the IκB proteins [45]. The NF-κB/Rel subunits then migrate into the nucleus, where they dimerise and induce the transcription of predominantly immuno-relevant target genes [46]. Included in this panel of target genes is the IκBα-encoding *NFKBIA* gene, which serves as part of an autocrine loop [47] to safeguard the oscillation of NF-κB between the nucleus and cytoplasm [47]. In general, the degradation and resynthesis of the various mammalian NF-κB inhibitors depend on adequate stimuli [48,49,50] and the presence of associated factors, such as the NF-κB-inhibitor-interacting Ras-like proteins (NKIRAS) [51,52].

Several members of the IκB family have been well conserved during evolution, as orthologs have been identified in birds [23], fishes [53], and even insects [54]. However, a detailed characterisation of the iκb proteins from lower vertebrates is still pending. Iκbα is the best researched iκb ortholog and has been characterised in different fish species [53,55,56,57,58,59,60,61,62,63,64], whereas its iκbβ paralog has apparently been lost in various bony fish families. According to the gene database of the National Center for Biotechnology Information (NCBI), the iκbβ-encoding *nfkbib* gene is absent from the sequenced genomes of the Salmonidae, Percidae, Gadidae, Carangidae, and Oryziinae, although it is still present in other teleost fish species from the Cyprinidae or Ictaluridae (as of December 2022). Iκbη encoded by the *ankrd42* gene is apparently absent from most of the teleostean genomes sequenced so far.

In this report, we provide evidence that iκbβ has not been lost from salmonid genomes, and we characterise this putative iκbβ together with the canonical iκbα and iκbε proteins, the nuclear iκbδ and iκbζ proteins, and bcl3 from the rainbow trout salmonid fish (*Oncorhynchus mykiss*). The findings of the present study, therefore, provide a comprehensive overview of the structural and functional diversity of nf-κb inhibitors in a non-mammalian model species while also offering starting points for further research into the inflammatory signalling processes in bony fish.

## 2. Results

### 2.1. Iκb Proteins from Rainbow Trout Are Encoded on 14 Distinct Genes

Our search of the NCBI gene database for *NFKBI* orthologs in the rainbow trout *Oncorhynchus mykiss* (assembly USDA_OmykA_1.1) yielded six *nfkbia*, two *nfkbie*, two *nfkbid*, two *nfkbiz*, and two *bcl3* genes (Table 1). By contrast, the orthologs of *NFKBIB* and *ANKRD42* seemed absent not only in salmonids but in many other teleost fishes. The same number of *nfkbi* genes was present in the closely related Chinook salmon, *Oncorhynchus tshawytscha* (assembly Otsh_v2.0) (Table 1, last column).

**Figure 1:**
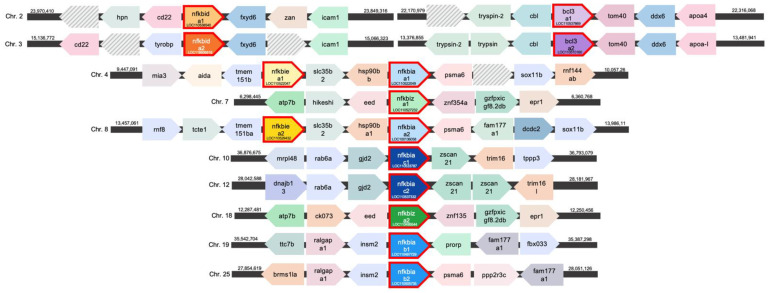
Synteny between the *nfkbi* genes in rainbow trout, *O. mykiss*, determined using the NCBI gene database. Arrows represent the reading direction of genes found in synteny; the same colours indicate orthologous/ohnologous genes. Numbers indicate the chromosomal location in nucleotides. Not characterised genes are represented by hatched boxes.

All *nfkbi* genes from rainbow trout are present as pairs of ohnologs that most likely arose from a whole-genome duplication, which is also reflected by their location on separate chromosomes [65,66]. Additionally, the *nfkbia* genes clearly exist as three pairs of duplicated paralogs (Figure 1): (a) one pair of ohnologous *nfkbia* genes (a1 and a2 on chromosomes 4 and 8, respectively) is located in the immediate vicinity of the genes *hsp90b* and *psma6*; (b) a second *nfkbia* pair (*b1* and *b2* on chromosome 19 and 25) both neighbour the gene *insm2* and lie in the vicinity of the gene *fam177a1*; and (c) the third *nfkbia* gene pair (on chromosomes 10 and 12) has been annotated adjacent to the genes *gjd2* and *zscan21*. The ohnologous *nfkbie* genes *a1* and *a2* are both in direct proximity to the genes *tmem15b* and *slc35b2*, located on the same chromosomes as the *nfkbia* genes *a1* and *a2*. The ohnologous *nfkbid* genes *a1* and *a2* on chromosomes 2 and 3 share *fxyd6* in their spatial vicinity, and the *bcl3* ohnologs on the same chromosomes are flanked by the genes *cbl* and *tom40*. The two *nfkbiz* ohnologs *a1* and *a2* on chromosomes 7 and 18 are adjacent to the genes *eed*, *znf*, and *epr1*.

The shorter coding sequences of *nfkbia* and *nfbkie* (both between 942 bp and 1191 bp in length) are distributed across 5 to 6 exons, while the coding sequences of *nfkbid* and *nfkbiz* are significantly longer (between 1461 bp and 1707 bp in length) and are divided over 9 to 12 exons. The *bcl3* ohnologs represent the longest *nfkbi* sequences (>2000 bp) in rainbow trout and are distributed across 12 exons. Based on automated computational analyses, the latest USDA_OmykA_1.1 assembly of the rainbow trout transcriptome includes one shorter *nfkbia* transcript variant and four shorter *nfkbiz* transcript variants, two of which are non-sense mRNAs (Table 2). These variants most likely arose from exon skipping during splice events, but neither this assumption nor the existence of the predicted transcript isoforms has been experimentally validated.

The sequence identity of the ohnologous *nfkbi*-encoded iκb proteins from rainbow trout (including bcl3) ranges from 82% to 100% (Table S1). The two pairs of the iκbα ohnologs a1/a2 and b1/b2 still share about 60% identity. However, comparison of the iκbα paralogs a1/a2 or b1/b2 versus c1/c2 reveals a sequence identity below 30%. This is a similarly low identity to that shared, for instance, by the paralogs iκbα and iκbε.

**Figure 2:**
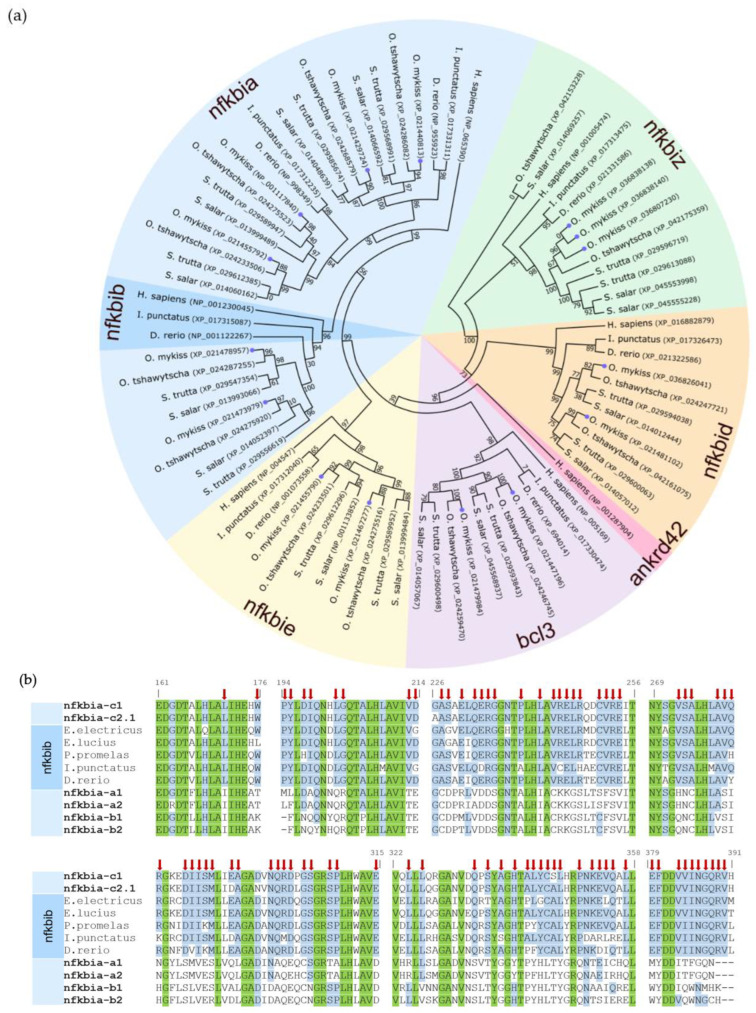
(**a**) Circular phylogenetic tree of selected IκB amino acid sequences from humans (*Homo sapiens*) and their orthologs in rainbow trout (*O. mykiss*; labelled with a purple dot at the branch end), Chinook salmon (*O. tshawytscha*), Atlantic salmon (*S. salar*), brown trout (*S. trutta*), channel catfish (*Ictalurus punctatus*) and zebrafish (*Danio rerio*); the NCBI accession codes are given in brackets. This neighbour-joining tree was constructed using the Poisson-correction distance model; bootstrap values are given at the nodes of each clade. The shaded underlays label the assignment to the IκB subfamilies iκbα/nfkbia (light blue), iκbβ/nfkbib (darker blue) iκbε/nfkbie (yellow), iκbδ/nkfbid (orange), iκbζ/nfkbiz (green), iκbη/ankrd42 (pink), and bcl3 (purple). (**b**) Alignment of various amino acid sections of the six putative iκbα/nfkbia sequences from rainbow trout and five selected iκbβ/nfkbib orthologs from electric eel (*Electrophorus electricus*; XP_026884418), northern pike (*Esox lucius*; XP_010862954), fathead minnow (*Pimephales promelas*; XP_039505967), channel catfish, and zebrafish. Green underlay marks amino acid residues that are identical across all selected sequences; blue underlay denotes those residues conserved between nfkbia-c sequences and at least two nfkbib sequences. Red arrows denote residues identical in at least one of the nfkbia-c ohnologs from trout and at least two nfkbib sequences from the other fish species, but different in at least two of the nfkbia-a and nfkbia-b sequences. Amino acid positions given above the alignment refer to the nfkbiac1 sequence.

A phylogenetic analysis across the amino acid sequences of all IκB proteins from humans and fishes revealed that the ohnologous iκbδ, iκbε, and bcl3 sequences from rainbow trout form separate clusters with their counterparts in the other salmonid fishes, including Chinook salmon *O. tshawytscha*, Atlantic salmon *Salmo salar*, and brown trout *Salmo trutta* (Figure 2a), whereas the iκbζ isoforms instead cluster in a species-specific fashion.

Unexpectedly, a pair of ohnologous iκbα sequences (c1 and c2) cluster with the human IκBβ factor (dark blue section on the left side of the dendrogram in Figure 2a), while the other two pairs of iκbα ohnologs (a1 and a2, as well as b1 and b2) cluster―as previously supposed―with the human IκBα factor. The homology of the iκbα ohnologs c1 and c2 with iκbβ from other teleost species is also reflected by 79 amino acid residues between positions 171 and 390 that are shared with the iκbβ sequences from at least two other fish species, but different in one or both iκbα-a and iκbα-b paralogous pairs (Figure 2b).

**Figure 3:**
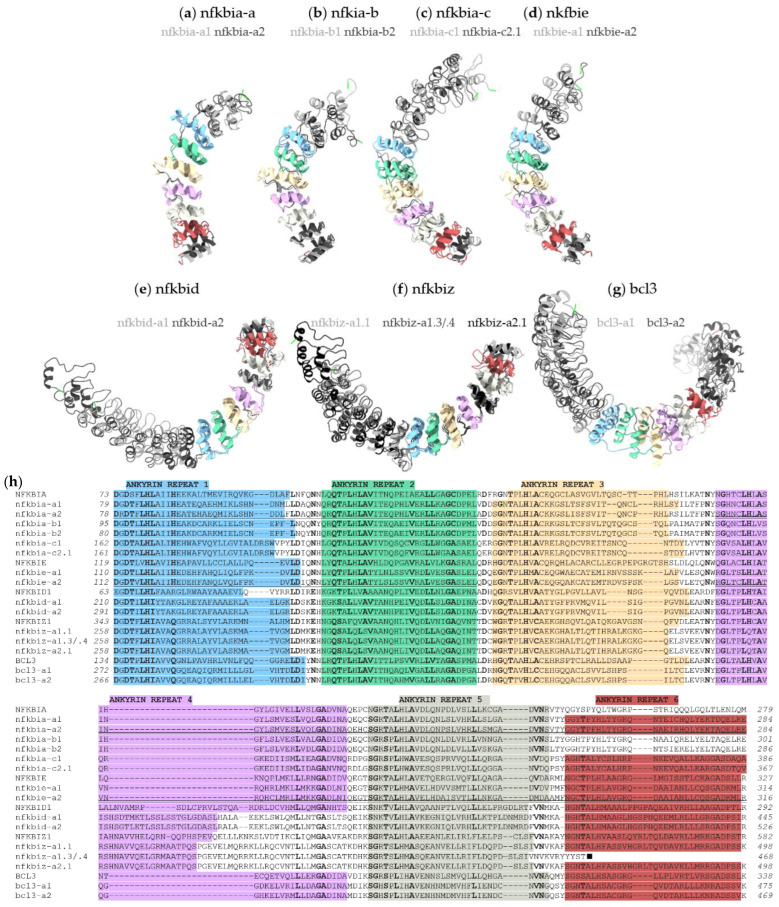
(**a**–**g**) Tertiary structures of the ohnologous iκb proteins from the rainbow trout (*O. mykiss*). One ohnolog is coloured in light grey, and the other one is coloured in dark grey (or black for nfkbiza-2.1) to indicate structural (dis)similarities. The individual ankyrin-repeat domains are each shown in different colours. The N- and C-termini of each protein are indicated by green and red stretches, respectively. (**h**) Alignment of the ankyrin-repeat domains of the iκb proteins from rainbow trout and their human orthologs. The six ankyrin-repeat domains are coloured according to the above 3D structures (**a**–**g**). Bold characters mark amino acid residues that are well conserved across the aligned IκB sequences. A single underline indicates the sequence used for the expression constructs ‘nfkbia-AR12’ and ‘nfkbie-AR12’, while a double underline indicates the sequence used for the expression constructs ‘nfkbia-AR456’ and ‘nfkbie-AR456’. The black square indicates the end of the protein sequences of nfkbiza-1.3 and nfkbiz-a1.4. (For the NCBI accession codes see Figure 2).

The overall structural differences of the iκb proteins from rainbow trout are depicted with three-dimensional models highlighting the well-conserved ankyrin-repeat motifs (Figure 3a–g). A defined number of ankyrin-repeat motifs is a characteristic of all IκB proteins. The iκb proteins listed in the NCBI database for rainbow trout contain six ankyrin repeats, except for two iκbα isoforms (nfkbia-b1, nfkbia-b2) and two iκbζ variants (nfkbiz-a1.3, nfkbiz-a1.4) (Figure 3h). The iκbζ variants a1.3 and a1.4 lack the 6th ankyrin repeat, while the amino acid sequence of the 6th ankyrin repeat of the iκbα isoforms b1 and b2 differs from the canonical motif. In addition, the sequences of the 4th ankyrin repeats of both the iκbδ and iκbζ proteins differ from their counterpart sequences in iκbα and iκbε as well as bcl3.

### 2.2. IκBα-Encoding nfkbia-a Transcripts Are Most Strongly Expressed in Immune Tissues and Immune-Cell Fractions

**Figure 4:**
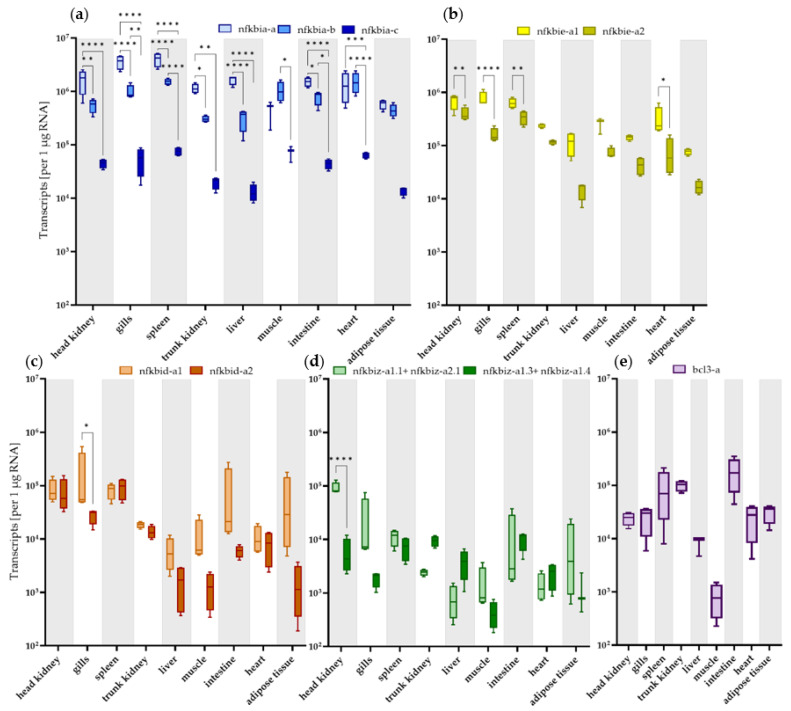
Levels of *nfkbi* transcripts per µg RNA in (**a**–**e**) various tissues (as listed on the abscissa) from the rainbow trout *O. mykiss*. Bars represent the averaged copy numbers (*n* = 3) normalised against the reference genes *eef1a1* and *rps5*; error bars represent the standard error of the mean (SEM). Asterisks represent significantly different transcript levels across transcript isoforms (*, *p* < 0.05; **, *p* < 0.01; ***, *p* < 0.001; ****, *p* < 0.0001).

Fifteen genes from rainbow trout produce (at least) seventeen transcript variants coding for iκbα, iκbε, iκbδ, iκbζ, or bcl3 (Table 1 and Table 2), as identified in the relevant gene database. We determined the transcript levels of the individual *nfkbi* genes in nine selected tissues and two sorted immune-cell fractions by designing primer pairs common to ohnologs of the *nfkbia-a*, *nfkbia-b*, *nfkbia-c*, and *bcl3* genes as well as primer pairs discriminating between the ohnologs of *nfkbie*, *nfkbid*, and *nfkbiz* (Table 3 and Table S2). Across the quantified *nfkbia* genes, the *nfkbia-a* transcripts had the highest levels in the spleen (4.1 × 10^6^ copies), gills (3. 6 × 10^6^ copies), head kidney, and trunk kidney (1.1 to 1.7 × 10^6^ copies) and exceeded the levels of the *nfkbia-b* ohnologs by 2.6- to 3.7-fold and the *nfkbia-c* ohnologs by 37- to 66-fold in the same four tissues (with *p* < 0.05) (Figure 4a).

In the liver, muscle, intestine, heart, and adipose tissue, the *nfkbia* expression differences were less pronounced and, in part, not significant. High transcript levels were also recorded for the *a1* ohnoloe of the *nfkbie* gene in the gills (7.6 × 10^5^ copies), head kidney (7.1 × 10^5^ copies), and spleen (6.3 × 10^5^ copies), and these levels exceeded those of the *nfkbie*-*a2* ohnologs by a factor of about 2 to 5 (with *p* < 0.01) (Figure 4b). The transcripts for the *nfkbid* and *nfkbiz* ohnologs ranged between about 430 (*nfkbiz-a1.3+1.4* in the muscle) and 1.7 × 10^5^ copies (*nfkbid-a1* in the gills) but showed few significant differences in transcript levels between the ohnologs (Figure 4c,d). With regard to tissue-specific expression patterns, the transcript levels of *nfkbia-a* and *nfkbie* were significantly higher in immune-relevant tissues, including head kidney, gill, and spleen, but (almost) no significant differential expression was observed for *nfkbia-c*, *nfkbid-a1*, *nfkbid-a2*, or *nfkbiz-a1.3+1.4* between tissues.

**Figure 5:**
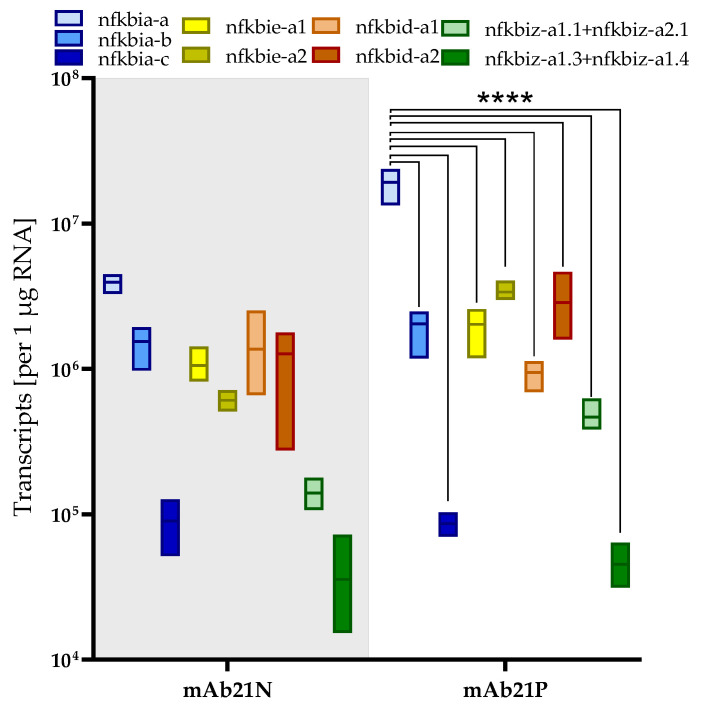
Levels of *nfkbi* transcripts per µg RNA in cell fractions from the rainbow trout (*O. mykiss*) (as listed on the abscissa). Bars represent the averaged copy numbers (*n* = 3) normalised against the reference genes *eef1a1* and *rps5*; error bars represent SEM. Asterisks represent significantly different transcript levels across different *nfkbi* genes (****, *p* < 0.0001).

The tissue-wise expression profiles suggested that organs rich in immune cells generally have high levels of *nfkbi* transcripts. For this reason, we quantified the *nfkbi* transcripts in (i) a non-myeloid (mAb21N) fraction enriched with T- and B-lymphocytes, natural killer-like cells, and thrombocytes and (ii) a myeloid (mAb21P) fraction enriched with granulocytes, monocytes/macrophages, and dendritic cells from the head kidney of the rainbow trout (*O. mykiss*) (Figure 5).

Again, the level of *nfkbia-a* transcripts was the highest (19 × 10^6^ and 4 × 10^6^ copies in the mAb21P and mAb21N fractions, respectively) compared to the other *nfkbi* transcripts, but this difference was only statistically significant for the mAb21P fraction (*p* < 0.0001). The levels of *nfkbiz-a1.3+1.4* were the lowest (3.6 × 10^4^ to 4.5 × 10^4^ copies in the mAb21P and mAb21N fractions, respectively).

### 2.3. Iκbα and iκbε Localise to the Cytoplasm as well as the Nucleus of Salmonid Model CHSE-214 Cells

Their prominent expression in the immune tissues of rainbow trout suggested that *nfkbia-a2* and *nfkbie-a2* were appropriate iκb factors to seek the first insights into the regulation of nf-κb pathways in salmonid fish.

**Figure 6:**
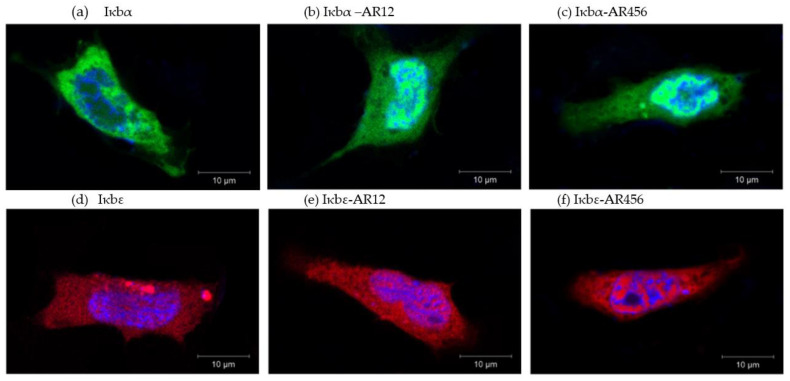
Overexpression of gfp-tagged iκbα (green fluorescence) or plum-tagged iκbε constructs (red fluorescence) in salmonid CHSE-214 cells. Confocal analysis of (**a**) iκbα, (**b**) ankyrin repeats 1 and 2 of iκbα, (**c**) ankyrin repeats 4, 5, and 6 of iκbα, (**d**) iκbε (red), (**e**) ankyrin repeats 1 and 2 of iκbε, and (**f**) ankyrin repeats 4, 5, and 6 of iκbε in CHSE-214. Nuclei were stained with Hoechst 33,342 dye (blue fluorescence); see Figure S1 for images without Hoechst staining. White scale bar represents 10 μm in all images.

The ankyrin repeat is the signature motif of all IκB proteins (cf. Figure 3). We transiently overexpressed each of the three construct variants of iκbα or iκbε in CHSE-214 cells, including (i) the full sequence as well as truncated variants comprising (ii) the two N-terminal ankyrin repeats and (iii) the three C-terminal ankyrin repeats (Figure 3h). Confocal imaging indicated a differential localisation of the different iκbα or iκbε constructs (Figure 6). The concentration of the full-length iκbα was higher in the cytoplasm than in the nucleus (Figure 6a), while both iκbα-AR12 and iκbα-AR456 proteins were localised to a greater extent in the nucleus than in the cytoplasm (Figure 6b,c). The full-length iκbε factor and its derivative iκbε-AR12 and iκbε-AR456 proteins seemed to be evenly distributed in both the cytoplasm and nucleus (Figure 6d–f).

### 2.4. Iκbα and iκbε Reduce the Basal and Stimulated nf-κb Activity

**Figure 7:**
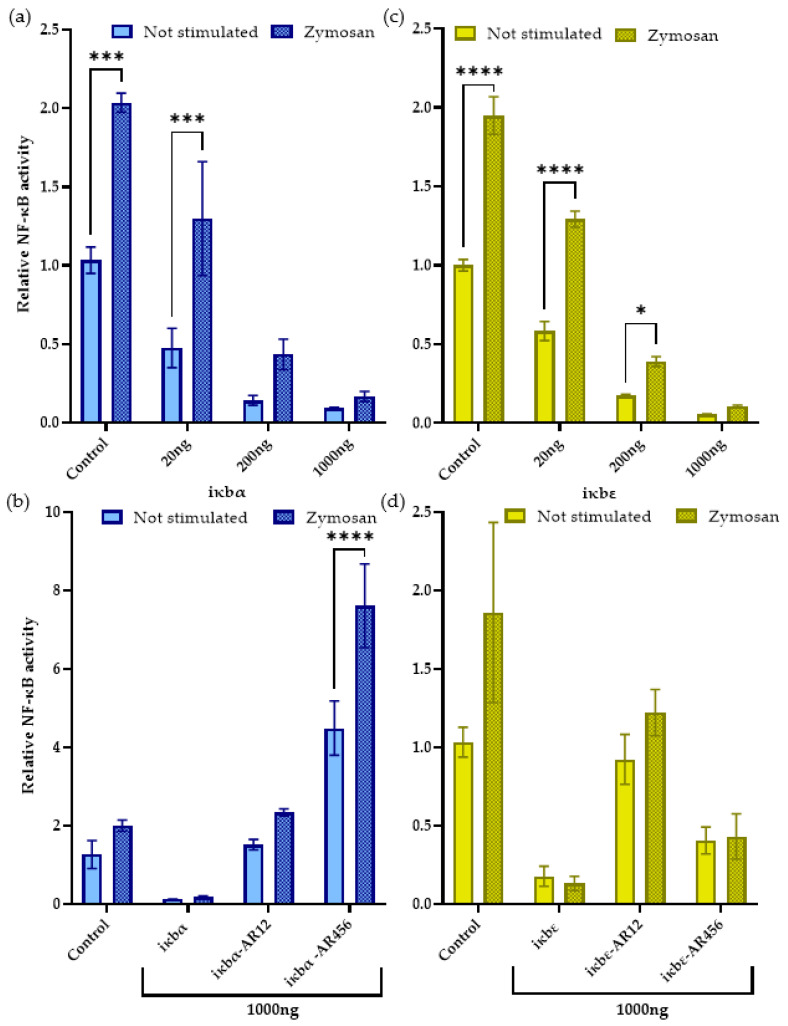
Overexpression of (**a**,**b**) gfp-tagged iκbα (green fluorescence) or (**c**,**d**) plum-tagged iκbε constructs (red fluorescence) in salmonid CHSE-214 cells. The luciferase activity of the ELAM-reporter vector was quantified in CHSE-214 cells co-expressing one of the six iκb constructs expressing (**a**,**b**) full-length iκbα and its truncated derivatives iκbα-AR12 and iκbα-AR456 and (**c**,**d**) full-length iκbε and its truncated derivatives iκbε-AR12 and iκbε-AR456. The concentrations of the iκb-expressing vector used for the transfection of the cells are indicated on the abscissa. Bars denote the mean values ± SEM. Statistical significance was assessed using two-way ANOVA (*, *p* < 0.05; ***, *p* < 0.001; ****, *p* < 0.0001).

We used the six iκbα or iκbε expression constructs described in Section 2.3 and Section 4.2 to assess their impact on the nf-κb activity in CHSE-214 cells. The overexpression of the full-length iκbα factor (1000 ng) robustly and significantly reduced the basal nf-κb activity down to 0.09-fold (*p* = 0.0004) (Figure 7a) compared to the non-transfected controls. Similarly, the overexpression of full-length iκbε (1000 ng) resulted in only 0.06-fold basal nf-κb activity (with *p* < 0.0001) compared to the controls (Figure 7b). In contrast to the full-length constructs, the two N-terminal ankyrin repeats or the three C-terminal ankyrin repeats of iκbα enhanced the basal nf-κb activity by 11.8-fold (*p* = 0.08) or 35.0-fold (*p* < 0.00001), respectively (Figure 7c). The pattern for the truncated iκbε constructs differed in the stronger nf-κb activation (5.2-fold; *p* = 0.23) caused by the N-terminal ankyrin repeats than by the C-terminal domains (2.3-fold; *p* = 0.79) (Figure 7d).

Stimulation of the non-transfected CHSE-214 cells with the fungal cell wall component zymosan doubled the nf-κb activity (2.0-fold; *p* < 0.0001) compared to the basal state (Figure 7a,b). Increasing the amounts of overexpressed iκbα factor from rainbow trout dose-dependently lowered this stimulated nf-κb activity down to 0.66-fold (20 ng expression vector; *p* < 0.05) and 0.05-fold (1000 ng expression vector; *p* < 0.001) compared to the non-transfected cells (Figure 7a). Again, the overexpression of iκbε had a very similar effect on the stimulated nf-κb activity, as observed for iκbα (*p* < 0.0001) (Figure 7b). Cells overexpressing either the N-terminal or the C-terminal ankyrin repeats of iκbα or iκbε showed an already enhanced basal nf-κb activity, and stimulation induced a further increase in active nf-κb.

**Figure 8:**
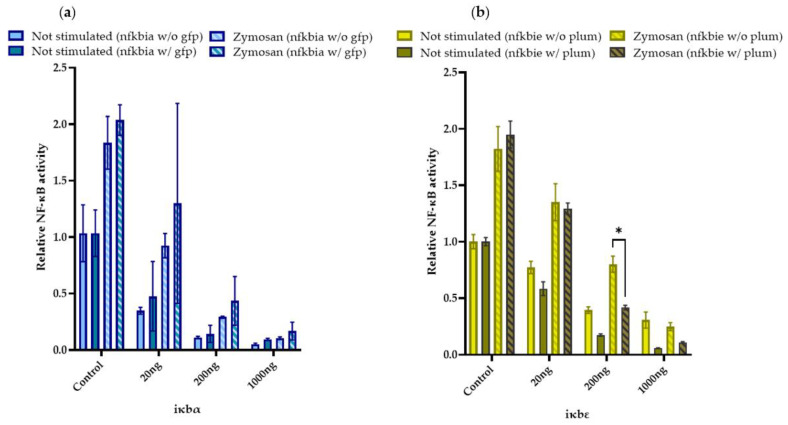
Overexpression of (**a**) green fluorescent gfp-tagged iκbα (w/gfp) and iκbα without gfp (w/o gfp) or (**b**) red fluorescent plum-tagged iκbε constructs (w/plum) and iκbε without plum (w/o plum) in salmonid CHSE-214 cells. The luciferase activity of the ELAM-reporter vector was quantified in CHSE-214 cells co-expressing the six iκb constructs. The concentrations of the iκb-expressing vector used for transfection of the cells are indicated on the abscissa. Bars denote the mean values ± SEM. Statistical significance was assessed using two-way ANOVA (*, *p* < 0.05).

Admittedly, the expression constructs used for the above overexpression studies did not only encode distinct iκb factors but also a fluorescent protein (gfp or plum). To exclude the possibility that the fluorescent protein had an additional effect on the nf-κb activity, we verified that expression vectors encoding either iκbα or iκbε coupled to a fluorescent protein (gfp or plum) and an expression plasmid encoding either iκbα or iκbε without fluorescent protein had a similar effect on the nf-κb cellular activity (Figure 8).

Notably, zymosan stimulation induced a significantly different nf-κb activity in CHSE-214 cells transfected with 200 ng iκbε tagged with plum versus iκbε without plum, but this differential activity pattern was not consistent across the other concentrations. Therefore, we do not assume any significant influence of the fluorescent tag on the performance of the expressed iκb factor.

**Figure 9:**
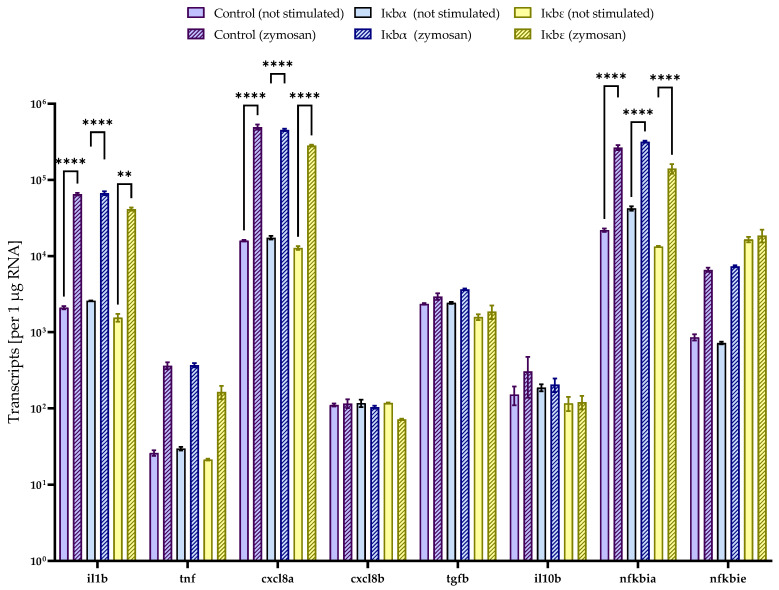
Expression profiling in CHSE-214 cells overexpressing iκbα and iκbε (representative of two experiments performed in triplicate). The bar chart illustrates the average number of specific transcripts (+SEM), as listed on the abscissa. Expression values were normalised against the geometric mean of the two reference genes. Transcript concentrations were quantified in unstimulated cells (unpatterned bars) and cells stimulated with zymosan for 4 h (striped), either transfected without (purple) or with iκbα- (blue) or iκbε-expression vectors (yellow). Statistical significance was assessed using two-way ANOVA (**, *p* < 0.01; ****, *p* < 0.0001).

Having established that both iκbα and iκbε factors from rainbow trout significantly reduced the nf-κb activity in vitro, we used qPCR to test whether the overexpression of both factors would modulate the transcription of a panel of nf-κb-dependent immune genes in the same CHSE-214 model cells. The transcript levels of the seven selected immune genes were similar between untransfected cells and cells overexpressing iκbα or iκbε (Figure 9).

Stimulation with zymosan for four hours significantly (*p* < 0.01) increased the transcript levels of characteristic inflammatory markers, such as il1b and cxcl8, but also of nfkbia (LOC112249975). Nevertheless, the differences in transcript levels of the induced immune genes in non-transfected cells versus cells expressing iκbα or iκbε were not statistically significant after stimulation with zymosan.

## 3. Discussion

Previous reports have suggested that rainbow trout possess one [53] or four [59] functional iκbα-encoding genes, but our research at the NCBI gene database revealed six *nfkbia* gene copies on different chromosomes of *O. mykiss*. Only one *NFKBIA* gene is present in mammals and two *nfkbia* paralogs have been characterised in several fish species, including zebrafish *D. rerio* [58], rock bream *Oplegnathus fasciatus* [55], orange-spotted grouper *Epinephelus coioides* [57], and blunt snout bream *Megalobrama amblycephala* [59]. It is rather unlikely that the additional teleost-specific whole-genome duplication in fish yielded three and not two *nfkbia* paralogs in salmonids, which then underwent an additional genome duplication [67] that eventually produced three pairs of *nfkbia* ohnologs. Several mechanisms could explain why certain genes are present in more copies than expected. For instance, the tandem duplication of genes arises from the unequal exchange between sister chromosomes [68]. Our structural and phylogenetic analyses suggest that one pair of the putative *nfkbia* ohnologs is the supposedly lost *nfkbib* gene. The sequence identity between the iκbα ohnologs a1/a2 or b1/b2 versus c1/c2 is comparably low, as reflected by our phylogenetic analysis that assigned the a1/a2 and b1/b2 pairs to the α-subfamily of IκB factors, while the c1/c2 pair was assigned to the β-subfamily of IκB factors. However, the genes flanking the human *NFKBIB* gene (including *SIRT2*, *RINL*, *SARS2*, and *CCR2*) [69] are either not present in rainbow trout or they are located on different chromosomes where they flank different genes. Conversely, the genes flanking *nfkbia-c* (presumably *nfkbib*) in rainbow trout (such as *gjd2*, *rab6a* and *zscan21*) are located on different chromosomes in the human genome, where they do not flank the same genes as they do in the trout genome. However, *nfkbib* genes are present in various teleost fishes, and *nfkbib* from the northern pike *Esox lucius*, for instance, is adjacent to the same genes as *nfkbia-c* (presumably *nfkbib*) in the rainbow trout genome (such as *rab6a* or *gjd1a*). The NCBI gene database (accessed on 14 December 2022) lists one *nfkbib* copy in 24 species, including 6 representatives of the Cypriniformes (carp fishes), 4 representatives of the Siluriformes (catfishes), 3 representatives each of the Characiformes (characins), and Clupeiformes (herring) and 2 representatives of the Osteoglossiformes (elephantfishes). In stark contrast, *nfkbia* and *nfkbie* are encoded in the genomes of more than 120 sequenced fish species, and *nfkbiz* is present in 115 species. The number of fish species possessing *nfkbid* is significantly lower at 63. We conclude from these indications that iκbβ has certainly been lost in many of the fish species sequenced thus far but not in salmonid fishes.

Three previous studies provide kaleidoscopic insights into the distinct characteristics and functions of teleostean iκbα paralogs: (a) iκbα-a is downregulated in the liver of the rock bream *O. fasciatus* a few hours after stimulation with flagellin, while its paralog iκbα-b is upregulated [55]; (b) the iκbα-a protein from the orange-spotted grouper *Epinephelus coioides* is distributed across the cytoplasm and nucleus, while its paralog iκbα-b mainly localises to the cytoplasm [57]; (c) the tnf-stimulated resynthesis of iκbα-b from the zebrafish *D. rerio* takes twice as long as the production of its paralog iκbα-a [58]. Our data on the iκbα paralogs and ohnologs of rainbow trout reveal that the expression of the iκbα-a-encoding gene is significantly higher in immune organs and in a head-kidney cell fraction enriched with granulocytes, monocytes/macrophages, and dendritic cells compared to its paralogs. Similar to what is observed in mammals, the iκb-encoding *nfkbi* genes from rainbow trout are constitutively expressed, albeit in a tissue-specific fashion [17,18,41]. These tissue-specific expression patterns obviously vary in different fish species, as demonstrated at least for *nfkbia*. The prominent *nfkbia* transcript level in the spleen of rainbow trout is in line with findings in the Japanese eel *Anguilla japonica* [63] and the mandarin fish *Siniperca chuatsi* [64], but it contrasts with the rather low splenic expression in the rock bream *O. fasciatus* [55], the blunt snout bream *M. amblycephala* [59] or the half-smooth tongue sole *Cynoglossus semilaevis* [60]. No comparable datasets are presently available for the expression profiles of the other *nfkbi* transcripts. In addition to the tissue-specific expression patterns, differential *nfkbia* transcript levels have been identified as indicative parameters for immune stimulation [70,71,72], exposure to toxic substances [73], or consumption of different diets [74,75] in diverse fish species, including rainbow trout [75,76]. The present study also confirms the significant upregulation of *nfkbia*, but not *nfkbie*, after in vitro fungal stimulation.

Iκbα and iκbε from rainbow trout localise to the cytoplasm and nuclei of unstimulated cells, as observed for IκBα orthologs from mammals [28] and bony fish [63]. Mammalian IκBε has been detected in the cytoplasm as well as in the nucleus [77], while the spatial distribution of iκbε in teleostean cells has not yet been analysed. In contrast to the full iκbα-protein from rainbow trout, the truncated iκbα versions were mostly located in the nucleus.

The overexpression of iκbα from rainbow trout blocked the basal and stimulated nf-κb activity in vitro, which is in line with many other reports on similar reporter-gene experiments in fish cells [56,57,63]. This again proves that the function of IκBα as an efficient regulator of NF-κB signalling is well preserved across vertebrates. The effectiveness of iκbε from rainbow trout was similar to that of iκbα in terms of restricting nf-κb activity, but no comparative data on the biological activity of its orthologue in other teleost species are presently available.

Our expression constructs encoding either the first two or the last three ankyrin repeat motifs of iκbα and iκbε from rainbow trout increased the nf-κb activity, whereas the full-length iκbα and iκbε proteins did not. Moreover, we observed that the truncated iκbα variants had higher concentrations in the nucleus than in the cytoplasm, whereas the opposite was apparent for their full-length counterparts. The two N-terminal ankyrin repeat domains of the mammalian IκBα are known to contact the nuclear localisation signal of rela/nf-κB p65 [35,78,79]. Thus, the N-terminal domains of iκbα from rainbow trout quite plausibly have significant involvements in the oscillations of nf-κB. The three C-terminal ankyrin repeat domains in the mammalian IκB ortholog interact with the N-terminal domain of the Rel homology region or the PEST region of rela/nf-κB p65 and/or nfkb1/nf-κB p50 [35,78,79]. Accordingly, the ankyrin repeat domains seem to fulfil specific functions in fish, as they do in mammals, and their number and position may be crucial for their ultimate function.

The number of ankyrin-repeat motifs is probably not a criterion that discriminates iκbα from iκbβ. Iκbαa from orange-spotted grouper *E. coioides* contains five ankyrin repeat motifs, while iκbαb contains six ankyrin repeats [57]. In rainbow trout, the paralog pairs iκbα-a and iκbα-c/iκbβ possess six ankyrin-repeat motifs, while iκbα-b contains only five prototypical ankyrin-repeat motifs. Although the ankyrin-repeat domain is the most conserved feature of IκB proteins, it is found in many other proteins. Therefore, we can assume that more ankyrin repeat-containing proteins affect the activity of NF-κB than are currently known. In mammals, the ankyrin repeat-containing proteins INK4 and myotrophin, for instance, have been proven to modulate the efficacy of NF-κB functions [80,81], but their role in inflammatory processes in fish is not yet known.

In summary, the intensive structural comparisons presented here demonstrate that iκbβ does exist in salmonid fish, but its expression is significantly lower than that of the paraloguous iκbα proteins. Our comprehensive overexpression studies in trout cells provide insights into the regulation potential of a set of nf-κb inhibitors from rainbow trout, thereby providing the first functional results for iκbε in lower vertebrates. In ongoing studies, we are investigating the interplay between nf-κb inhibitors and nf-κb/rel factors from trout under defined environmental conditions.

## 4. Materials and Methods

### 4.1. Quantitative PCR (qPCR) Analysis

We recorded the expression of *nfkbia-a*, *nfkbia-b*, *nfkbi-c*, *nfkbie*, *nfkbid*, and *nfkbiz* in nine tissues (adipose tissue, gills, head kidney, heart, intestine, liver, muscle, spleen and trunk kidney) and sorted cells from rainbow trout. All analyses were conducted using animal materials left over from previous analyses [82]. We used the monoclonal antibody mAb21 to separate an mAb21-positive head-kidney cell fraction consisting of >95% myeloid cells from a mAb21-negative fraction consisting mostly of B- and T-lymphocytes, as well as thrombocytes. RNA was isolated from tissues and sorted cells using the RNeasy Mini Kit (Qiagen, Hilden, Germany) and the ISOLATE II RNA Micro Kit (Bioline/Meridian Bioscience, Luckenwalde, Germany), respectively, including an in-column DNase treatment. We also profiled the expression of various Chinook salmon-specific immune genes (*il1b*, *tnf*, *cxcl8a*, *cxcl8b*, *tgfb*, *il10*, *nfkbia* and *nfkbie*) in transfected, stimulated CHSE-214 cells (derived from Chinook salmon [*O. tshawytscha*] embryos). After stimulation, the transfected CHSE-214 cells were washed twice with phosphate-buffered saline and then harvested by a 20-min incubation in lysis buffer (RNeasy Mini Kit; Qiagen, Hilden, Germany).

After isolation, RNA was reverse transcribed into cDNA using the SensiFAST cDNA Synthesis Kit (Bioline/Meridian Bioscience). Subsequently, the quantity of the *nfkbi* transcripts was selectively recorded by a panel of exon-skipping oligonucleotide primers specific for rainbow trout (Pyrosequencing Assay Design software v.1.0.6; Biotage, Uppsala, Sweden; Table 2). These primers were either common for both *nfkbi* ohnologs (*nfkbia-a*, *nfkbia-b nfkbi-c*, and *bcl3-a*) or discriminated between *nfkbi* ohnologs (*nfkbie*, *nfkbid*, and *nfkbiz*). Of particular note, no discriminating primers could be designed for the individual *nfkbiz* gene variants a1 and a2 due to the high sequence identity (99–100%) (Table S1); instead, we derived common primers for each of the two similar *nfkbiz* transcript variants *nfkbiz-a1.1/a2.1* and *nfkbiz-a1.3/a1.4*. The primer pairs listed in Table 3 amplified fragments between 86 and 191 nucleotides in length. *Rps5* (ribosomal protein S5) and *eef1a1* (eukaryotic translation elongation factor) were used as reference genes to normalise the expression data. The qPCR analyses were conducted using the LightCycler-96 system (Roche, Basel, Switzerland) according to the following programme: initial denaturation at 95 °C for 5 min, followed by 40 cycles of denaturation at 95 °C for 30 s, primer annealing at 60 °C for 15 s, elongation at 72 °C for 15 s, and the fluorescence measurement at 72 °C for 10 s. The amplicon quality was assessed by gel electrophoresis and melting-curve analysis. In addition, we checked the primer specificity by sequencing the amplicons (Azenta Life Sciences, Griesheim, Germany). The qPCR data were extracted using the LightCycler-96 analysis software v. 1.1.0.1320 (Roche).

### 4.2. Construction of Nfkbi-Expression Constructs

Three different vectors were used to express the complete ORFs of selected *nfkbi* genes (*nfkbia* and *nfkbie*) or distinct fragments of those genes (i.e., ankyrin repeat 1 and 2 or ankyrin repeat 4, 5, and 6) from rainbow trout. These vectors included the mammalian expression vector v280 [83] and two v280 derivatives attaching the red fluorescent protein mPlum or a green fluorescent protein (gfp) at the 3′ end of the inserted target fragments. These fragments were generated using Platinum Taq High-Fidelity DNA polymerase (Thermo Fisher Scientific, Bremen, Germany) and oligonucleotide primers linked with specific restriction sites (Table 4).

The Biometra TAdvanced cycler (Analytik Jena, Jena, Germany) was used to amplify the gene fragments according to the following programme: initial denaturation at 94 °C for 2 min, followed by 40 cycles of denaturation at 94 °C for 30 s, primer annealing at 60 °C for 30 s, elongation at 72 °C for 2 min, and a final extension step at 68 °C for 5 min. Amplicons were inserted into the above expression vectors by double digestion with *Hind*III and *EcoR*I (*nfkbia*) or *Bgl*II (*nfkbie*). All expression vectors were sequenced and checked for correct assembly before use.

### 4.3. Cell Transfection, Luciferase Assay and Confocal Microscopy

The salmonid cell line CHSE-214 was cultured as described previously [83]. The CHSE-214 cells were transfected in six-well plates with a total of 2050 ng endotoxin-free prepared DNA (ZymoPure II Plasmid Maxi Prep Kit, ZymoResearch, Freiburg, Germany) using X-tremeGENE HP DNA Transfection Reagent (Roche, Mannheim, Germany). The co-transfection assays contained 50 ng of the NF-κB-responsive promoter (endothelial-leukocyte adhesion molecule)-reporter (luciferase) construct ELAM-1-luc, defined concentrations (20 ng–1000 ng) of *nfkbi* expression vectors, and varying amounts of empty-vector DNA to ensure that the total DNA amount per assay remained constant.

For the stimulation experiments, co-transfected cells were split into 24-well plates: Three wells per row remained unstimulated, while the other three wells were challenged with 1 mg/mL zymosan from *Saccharomyces cerevisiae* (tlrl-zyn; Invivogen, Toulouse, France) for 4 h or 24 h. After incubation, the cell lysates were collected, and the luciferase activity of each assay was measured using the Dual-Luciferase Reporter Assay System (Promega, Mannheim, Germany) at the Lumat LB9501 luminometer (Berthold, Bad Wildbad, Germany). The resulting relative light units were normalised by the protein concentrations of the CHSE-214 cell extracts. Each transfection experiment was measured in triplicate and conducted at least twice.

The IκBα and IκBε factors were localised by transfecting CHSE-214 cells with different vectors expressing *nfkbia* and *nfkbie* tagged with green fluorescent protein (gfp) or plum, respectively. Hoechst 33342 dye (250 µg/mL; Sigma-Aldrich/Merck, Hamburg, Germany) was used to stain the nuclei 30 min before fixation of the CHSE-214 cells with 4% paraformaldehyde (Merck KGaA, Darmstadt, Germany). The cells were then examined with confocal microscopy (LSM 780; Carl Zeiss Microscopy, Oberkochen, Germany), using a 63× oil-immersion differential interference contrast objective.

### 4.4. Data Analysis

The qPCR data was normalised against the reference genes and based on gene-specific standard curves, and the individual copy numbers were calculated (R^2^ > 0.99; 10^7^–10^3^ copies per 5 µL). The GraphPad Prism software (v9.1.0) was used for the statistical analysis of the normalised qPCR data. Significant differences between the different tissues/cell fractions were assessed using two-way analysis of variance (ANOVA) followed by a Holm-Šídák’s post-hoc test to correct for multiple comparisons. A parametric *t*-test conducted using GraphPad Prism software v.9.5.1 was run to evaluate the statistical significance of the reporter-gene measurements. *p*-values less than 0.05 were considered statistically significant.

Orthologous *NFKBI* gene sequences were retrieved from the NCBI gene database. The protein sequence identity was determined by using https://npsa-prabi.ibcp.fr/NPSA/npsa_clustalw.html (accessed on 16 January 2023).

The ClustalW alignment tool [84] was used to align the NFKBI amino acid sequences. The phylogeny of orthologous IκB proteins was assessed using ETE3 on the GenomeNet (https://www.genome.jp/tools/ete/, accessed on 3 January 2023) [85].

A phylogenetic dendrogram was reconstructed with the neighbour-joining method based on log-corrected distances and optimised manually. Node robustness was evaluated on a bootstrap analysis based on 1000 iterations. SMART (Simple Modular Architecture Research Tool) [86] was applied to identify motifs and domains of the IκB proteins. The three-dimensional iκb protein structures were predicted using I-TASSER (Iterative Threading ASSEmbly Refinement) [87] in complement with UCSF ChimeraX v.1.1 [88].

## Figures and Tables

**Table 1:** *Nfkbi* genes identified in rainbow trout *O. mykiss*.

Gene	Chromosome	LOC Symbol	CDS Length [nt]	UTR Length [bp]		Polyadenylation Motifs ^a^	Instability Motifs	Prot. NCBI Acc.#	Protein Length [aa]	Ankyrin Repeats	Orthologs in Chinook Salmon
				5′	3′						
*nfkbia-a1*	4	LOC110522049	942	91	368	4	4	XP_021455792	313	6	LOC112217446
*nfkbia-a2*	8	LOC100136058	945	49	407	4	2	NP_001117840	314	6	LOC112249975
*nfkbia-b1*	19	LOC110497729	1005	66	454	3	8	XP_021429724	334	5	LOC112244922
*nfkbia-b2*	25	LOC110505735	963	75	438	4	8	XP_021440813	320	5	LOC112256803
*nfkbia-c1*	10	LOC110533787	1176	403	1408	9	8	XP_021473979	391	6	LOC112250205
*nfkbia-c2*	12	LOC110537332	1191	425	1420	9	13	XP_021478957	396	6	LOC112257706
*nfkbie-a1*	4	LOC110522047	1059	392	885	8	16	XP_021455790	352	6	LOC112217444
*nfkbie-a2*	8	LOC110529432	1065	449	1421	5	14	XP_021467277	354	6	LOC112249971
*nfkbid-a1*	2	LOC110538540	1461	117	721	2	2	XP_021481102	486	6	LOC112245876
*nfkbid-a2*	3	LOC118936618	1707	536	797	1	2	XP_036826041	568	6	LOC112227108
*nfkbiz-a1*	7	LOC110527232	1605	106	1369	4	8	XP_036838138	534	6	LOC112247547
*nfkbiz-a2*	18	LOC110495544	1605	108	1369	4	8	XP_036807230	534	6	LOC112266936
*bcl3-a1*	2	LOC110537869	2061	444	380	7	9	XP_021479984	686	6	LOC112235280
*bcl3-a2*	3	LOC110510160	2010	1728	414	8	11	XP_021447196	669	6	LOC112226570

^a^ AATAAA and ATTAAA.

**Table 2:** Predicted *nfkbi*-transcript isoforms from *O. mykiss* recorded in the NCBI database.

Nfkbi Transcript Isoform	Chromosome	Nucleotide NCBI Acc.#	LOC Symbol	CDS Length [nt]	UTR Length [bp]		Polyadenylation Motifs ^a^	Instability Motifs	Prot. NCBI Acc.#	Protein Length [aa]	Ankyrin Repeats
					5′	3′					
nfkbia-c2.2	12	XM_021623283	LOC110537332	1101	425	1420	9	13	XP_021478958	366	6
nfkbiz-a1.2	7	XR_005052489	LOC110527232				None	0	No CDS		
nfkbiz-a1.3	7	XM_036982244	LOC110527232	1407	107	555	None	0	XP_036838139	468	5
nfkbiz-a1.4	7	XM_036982245	LOC110527232	1407	107	524	None	0	XP_036838140	468	5
nfkbiz-a2.2	18	XR_005037322	LOC110495544				None	0	No CDS		

^a^ AATAAA and ATTAAA.

**Table 3:** Primers used in this study for quantitative PCR analysis.

Gene	Primer Sequence 5′→3′ (Sense, Antisense)	NCBI-Nucleotide Accession Code	Fragment Length [bp]
*nfkbia-a*	GCATGTCTGATGATGAACAGATG, GAACTCCAGGTCCCAGAAGCC	XM_021600117, NM_001124368 ^b^	149
*nfkbia-b*	ACCCAGCTCCCAGCCATTATG, GACATCGATGCACAGGAGCAG	XM_021574049 ^a^, XM_021585138	135
*nfkbia-c*	GGGAGCTGAGGCAGGACTGT, CAACTACTCGGGGGTGAGTGC	XM_021618304, XM_021623282 ^a^, XM_021623283 ^a^	91
*nfkbid-a1*	AGTCAGCCGTATCATCTATGTTTT, CTTTATGTAGGCCGTTTGTGATC	XM_036970146	153
*nfkbid-a2*	AGGTTGAATCCAGACATCTGTAC, AATAATGGCTAGCTAGTAATGAGC	XM_021625427	191
*nfkbie-a1*	CTGTAGGGTTATTTATCGTTGTTG, ATTCTCTGCTAGCAAAGTGGTAC	XM_021600115	107
*nfkbie-a2*	GCAACCGCTACCTTTGGTTTCA, CGGTCAAGACTACCTGGAGTG	XM_021611602	140
*nfkbiz-a1.1*, *nfkbiz-a2.1*	TCGTCAATGTCAAGGCATTCAGT, AAGAACCTGGAGAATGAGCAGC	XM_036982243, XM_036951335	144
*nfkbiz-a1.3*, *nfkbiz-a1.4*	TTCTGAGCTGACAAACAGTGTTC, ACTAAACCCTCTAACATGAGTTCT	XM_036982244, XM_036982245	86
*bcl3-a*	GCCAGTCGTACAGTGGGAACA, CAAGAACAAGAWGGTAACAGATGT	XM_021624309, XM_021591521	160
*cxcl8a*	CCATTACTGAGGGGATGAGTCTG, GAGACACTGAGATCATTGCCACTC	XM_021625342 ^a^, XM_024415648	153
*cxcl8b*	CTACATGATACAAGGGAGAGG, GGAAGAAGTCATTGTCACAC	XM_036989276, XM_024434566	146
*il1b*	GCTGTGGAAGAACATATAGTGTTGG, GCTACCACAAAGTGCATTTG	XM_036979104, XM_024418276	198
*il10*	ATGAACAACAGAACACAGAACAACA, CCAATGTAGGAACTACTTCTCCTG	NM_001245099 ^a^, NM_001246350 ^c^, XM_042324963	113
*nfkbia*	GCACAGGAACAATGTAGCG, GATGAACAGATGTACGATGACATTAC	XM_021600117, XM_024377738	281
*nfkbie*	AGGAGCGGTTGGATTCTGCTTAT, CCTTCTCACCACCATCACTGAA	XM_021600115 ^b^, XM_021611602, XM_024419748, XM_024377733 ^a^	158
*tgfb*	CATTCCAAGGTGCTAGGTCTGT, ACATCGGCAAGACCCCCAAGA	XM_024386204, XM_024402049 ^a^, XM_021563342, XM_021596503 ^a^	121
*tnf*	TTTACCTGGCACTCCAAGGATC, GCATACCCTGAGACAACTCTCT	XM_024407165, XM_021572765 ^c^, XM_036971683 ^c^	93

^a^ Indicates one mismatch of the primer sequence in comparison with the CDS sequence. ^b^ Indicates two mismatches of the primer sequence in comparison with the CDS sequence. ^c^ Indicates at least two mismatches of the primer sequence in comparison with the CDS sequence.

**Table 4:** Primers used in this study for the development of *nfkbi*-expression constructs.

Gene/Construct Name	Primer Sequence 5′→3′ (Sense, Antisense)	NCBI-Nucleotide Accession Code	Fragment Length [bp]
*nfkbia*	CCCAAGCTTGATATGGATGTTTATAGAGTTTCAAACG ^a^, GATGACATTACATTTGGTCAGAATGAATTCTCAAC ^a^	NM_001124368 (position 50–991)	960
*nfkbia*-AR12	CCCAAGCTTATGGATGTTTATAGAGTTTCAAACG ^a^, GTGACCCGCGGATAGCAGACGAATTCGGG ^a^	NM_001124368 (position 50–493)	465
*nfkbia*-AR456	CCCAAGCTTATGAGCGGACACAACTGCCTC ^a^, GATGACATTACATTTGGTCAGAATGAATTCGGG ^a^	NM_001124368 (position 611–991)	402
*nfkbie*	CCCAAGCTTCTGATATGCAAAGCGCCGAAGATGCG ^a^, CCCGAATTCCTGATCAGAATGGCCCTCCAACCAC ^a^	XM_021611602 (position 450–1511)	1080
*nfkbie*-AR12	CCCAAGCTTATGCAAAGCGCCGAAGATGCG ^a^, GGGGCCAGCCTGGAGCTGAGATCTGGG ^a^	XM_021611602 (position 450–977)	546
*nfkbie*-AR456	CCCAAGCTTATGAGAGGTCTCACCTGTCTC ^a^, TCAGTGGTTGGAGGGCCATTCAGATCTGGG ^a^	XM_021611602 (position 1104–1511)	429

^a^ Underlining marks the attached sequences (composed of the restriction site, optionally a start codon and three additional nucleotides at the 5′-end).

## Data Availability

The qPCR and reporter-gene data generated during the current study are not publicly available but are available on request. The nucleotide and amino acid sequences of the analysed IκB factors are available in the NCBI database; the respective accession numbers are provided in the manuscript.

## Supplementary Materials

The following supporting information can be downloaded at: https://www.mdpi.com/article/10.3390/ijms241210229/s1.

## Abbreviations

Aa, amino acid(s); bcl3, B-cell chronic lymphatic leukemia protein 3; CDS, coding sequence; CHSE, Chinook salmon embryo; cxcl8, C-X-C motif chemokine ligand 8; eef1a1, eukaryotic translation elongation factor; ELAM, endothelial cell-leukocyte adhesion molecule; gfp, green fluorescent protein; IKK, IκB kinase; IκB, inhibitor of NF-κB; il1b, interleukin-1 beta; il10, interleukin-10; mAb21N, non-myeloid fraction enriched with T- and B-lymphocytes, natural killer-like cells and thrombocytes; mAb21P, myeloid fraction enriched with granulocytes, monocytes/macrophages and dendritic cells; NF-κB, nuclear factor kappa-light-chain-enhancer of activated B cells; NLS, nuclear translocation signal; nt, nucleotide(s); PAMP, pathogen-associated molecular pattern; PEST, proline (P), glutamate (E), serine (S), threonine (T); RHD, Rel homology domain; rps5, ribosomal protein S5; tgfb, transforming growth factor-β; tnf, tumour necrosis factor; WGD, whole-genome duplication.

## References

[1] Visvanathan K.V., Goodbourn S. (1989). Double-stranded RNA Activates Binding of NF-kappa B to an Inducible Element in the Human Beta-interferon Promoter. EMBO J..

[2] Sen R., Baltimore D. (1986). Inducibility of κ Immunoglobulin Enhancer-Binding Protein NF-κB by a Posttranslational Mechanism. Cell.

[3] Hayden M.S., Ghosh S. (2011). NF-ΚB in Immunobiology. Cell Res..

[4] Dorrington M.G., Fraser I.D.C. (2019). NF-ΚB Signaling in Macrophages: Dynamics, Crosstalk, and Signal Integration. Front. Immunol..

[5] Baeuerle P.A., Henkel T. (1994). Function and Activation of NF-KappaB in the Immune System. Annu. Rev. Immunol..

[6] Sha W.C., Liou H.C., Tuomanen E.I., Baltimore D. (1995). Targeted Disruption of the P50 Subunit of NF-ΚB Leads to Multifocal Defects in Immune Responses. Cell.

[7] Alcamo E., Mizgerd J.P., Horwitz B.H., Bronson R., Beg A.A., Scott M., Doerschuk C.M., Hynes R.O., Baltimore D. (2001). Targeted Mutation of TNF Receptor I Rescues the RelA-Deficient Mouse and Reveals a Critical Role for NF-ΚB in Leukocyte Recruitment. J. Immunol..

[8] Franzoso G., Carlson L., Poljak L., Shores E.W., Epstein S., Leonardi A., Grinberg A., Tran T., Scharton-Kersten T., Anver M. (1998). Mice Deficient in Nuclear Factor (NF)-κB/P52 Present with Defects in Humoral Responses, Germinal Center Reactions, and Splenic Microarchitecture. J. Exp. Med..

[9] Diamond G., Kaiser V., Rhodes J., Russell J.P., Bevins C.L. (2000). Transcriptional Regulation of β-Defensin Gene Expression in Tracheal Epithelial Cells. Infect. Immun..

[10] Baeuerle P.A., Baltimore D. (1988). I Kappa B: A Specific Inhibitor of the NF-Kappa B Transcription Factor. Science.

[11] DiDonato J.A., Hayakawa M., Rothwarf D.M., Zandi E., Karin M. (1997). A Cytokine-Responsive IκB Kinase That Activates the Transcription Factor NF-κB. Nature.

[12] Rebl A., Goldammer T. (2018). Under Control: The Innate Immunity of Fish from the Inhibitors’ Perspective. Fish Shellfish Immunol..

[13] Inoue J.-i., Kerr L.D., Kakizuka A., Verma I.M. (1992). IκBγ, a 70 Kd Protein Identical to the C-Terminal Half of P110 NF-ΚB: A New Member of the IκB Family. Cell.

[14] Rice N.R., MacKichan M.L., Israël A. (1992). The Precursor of NF-ΚB P50 Has IκB-like Functions. Cell.

[15] Zabel U., Baeuerle P.A. (1990). Purified Human IκB Can Rapidly Dissociate the Complex of the NF-κB Transcription Factor with Its Cognate DNA. Cell.

[16] Kerr L.D., Duckett C.S., Wamsley P., Zhang Q., Chiao P., Nabel G., Baeuerle P.A., Verma I.M. (1992). The Proto-Oncogene B CL-3 Encodes an IKB Protein. Genes Dev..

[17] Li Z., Nabel G.J. (1997). A New Member of the I KappaB Protein Family, I KappaB Epsilon, Inhibits RelA (P65)-Mediated NF-KappaB Transcription. Mol. Cell. Biol..

[18] Yamazaki S., Muta T., Takeshige K. (2001). A Novel IκB Protein, IκB-ζ, Induced by Proinflammatory Stimuli, Negatively Regulates Nuclear Factor-κB in the Nuclei. J. Biol. Chem..

[19] Fiorini E., Schmitz I., Marissen W.E., Osborn S.L., Touma M., Sasada T., Reche P.A., Tibaldi E.V., Hussey R.E., Kruisbeek A.M. (2002). Peptide-Induced Negative Selection of Thymocytes Activates Transcription of an NF-ΚB Inhibitor. Mol. Cell.

[20] Yamauchi S., Ito H., Miyajima A. (2010). IκBη, a Nuclear IκB Protein, Positively Regulates the NF-ΚB-Mediated Expression of Proinflammatory Cytokines. Proc. Natl. Acad. Sci. USA.

[21] Scheinman R.I., Beg A.A., Baldwin A.S. (1993). NF-Kappa B P100 (Lyt-10) Is a Component of H2TF1 and Can Function as an I Kappa B-like Molecule. Mol. Cell. Biol..

[22] Naumann M., Wulczyn F.G., Scheidereit C. (1993). The NF-ΚB Precursor P105 and the Proto-Oncogene Product Bcl-3 Are IκB Molecules and Control Nuclear Translocation of NF-ΚB. EMBO J..

[23] Davis N., Ghosh S., Simmons D.L., Tempst P., Liou H.C., Baltimore D., Bose H.R. (1991). Rel-Associated Pp40: An Inhibitor of the Rel Family of Transcription Factors. Science.

[24] Liou H.C., Nolan G.P., Ghosh S., Fujita T., Baltimore D. (1992). The NF-ΚB P50 Precursor, P105, Contains an Internal IκB-like Inhibitor That Preferentially Inhibits P50. EMBO J..

[25] Nolan G.P., Fujita T., Bhatia K., Huppi C., Liou H.C., Scott M.L., Baltimore D. (1993). The Bcl-3 Proto-Oncogene Encodes a Nuclear I Kappa B-like Molecule That Preferentially Interacts with NF-Kappa B P50 and P52 in a Phosphorylation-Dependent Manner. Mol. Cell. Biol..

[26] Beg A.A., Ruben S.M., Scheinman R.I., Haskill S., Rosen C.A., Baldwin A.S.J. (1992). I Kappa B Interacts with the Nuclear Localization Sequences of the Subunits of NF-Kappa B: A Mechanism for Cytoplasmic Retention. Genes Dev..

[27] Whiteside S.T., Epinat J.C., Rice N.R., Israël A. (1997). I Kappa B Epsilon, a Novel Member of the IκB Family, Controls RelA and CRel NF-ΚB Activity. EMBO J..

[28] Huang T.T., Kudo N., Yoshida M., Miyamoto S. (2000). A Nuclear Export Signal in the N-Terminal Regulatory Domain of IkappaBalpha Controls Cytoplasmic Localization of Inactive NF-KappaB/IkappaBalpha Complexes. Proc. Natl. Acad. Sci. USA.

[29] Bours V., Franzoso G., Azarenko V., Park S., Kanno T., Brown K., Siebenlist U. (1993). The Oncoprotein Bcl-3 Directly Transactivates through ΚB Motifs via Association with DNA-Binding P50B Homodimers. Cell.

[30] Hirotani T., Lee P.Y., Kuwata H., Yamamoto M., Matsumoto M., Kawase I., Akira S., Takeda K. (2005). The Nuclear IκB Protein IκBNS Selectively Inhibits Lipopolysaccharide-Induced IL-6 Production in Macrophages of the Colonic Lamina Propria. J. Immunol..

[31] Kuwata H., Matsumoto M., Atarashi K., Morishita H., Hirotani T., Koga R., Takeda K. (2006). IκBNS Inhibits Induction of a Subset of Toll-like Receptor-Dependent Genes and Limits Inflammation. Immunity.

[32] Motoyama M., Yamazaki S., Eto-Kimura A., Takeshige K., Muta T. (2005). Positive and Negative Regulation of Nuclear Factor-κB-Mediated Transcription by IκB-ζ, an Inducible Nuclear Protein. J. Biol. Chem..

[33] Massoumi R., Chmielarska K., Hennecke K., Pfeifer A., Fässler R. (2006). Cyld Inhibits Tumor Cell Proliferation by Blocking Bcl-3-Dependent NF-κB Signaling. Cell.

[34] Lux S.E., John K.M., Bennettt V. (1990). Analysis of EDNA for Human Erythrocyte Ankyrin Indicates a Repeated Structure with Homology to Tissue-Differentiation and Cell-Cycle Control Proteins. Nature.

[35] Huxford T., Huang D.B., Malek S., Ghosh G. (1998). The Crystal Structure of the IκBα/NF-κB Complex Reveals Mechanisms of NF-ΚB Inactivation. Cell.

[36] Kieran M., Blank V., Logeat F., Vandekerckhove J., Lottspeich F., Le Bail O., Urban M.B., Kourilsky P., Baeuerle P.A., Israël A. (1990). The DNA Binding Subunit of NF-κB Is Identical to Factor KBF1 and Homologous to the Rel Oncogene Product. Cell.

[37] Mercurio F., Didonato J., Rosette C., Karin M. (1992). Molecular Cloning and Characterization of a Novel Rel/NF-ΧB Family Member Displaying Structural and Functional Homology to NF-ΧB P50/P105. DNA Cell Biol..

[38] Michel F., Soler-Lopez M., Petosa C., Cramer P., Siebenlist U., Müller C.W. (2001). Crystal Structure of the Ankyrin Repeat Domain of Bcl-3: A Unique Member of the IκB Protein Family. EMBO J..

[39] Beg A.A., Baldwin A.S. (1993). The I Kappa B Proteins: Multifunctional Regulators of Rel/NF-Kappa B Transcription Factors. Genes Dev..

[40] Ernst M.K., Dunn L.L., Rice N.R. (1995). The PEST-like Sequence of I Kappa B Alpha Is Responsible for Inhibition of DNA Binding but Not for Cytoplasmic Retention of c-Rel or RelA Homodimers. Mol. Cell. Biol..

[41] Thompson J.E., Phillips R.J., Erdjument-Bromage H., Tempst P., Ghosh S. (1995). IκB-β Regulates the Persistent Response in a Biphasic Activation of NF-κB. Cell.

[42] Mathes E., O’Dea E.L., Hoffmann A., Ghosh G. (2008). NF-ΚB Dictates the Degradation Pathway of IκBα. EMBO J..

[43] McKinsey T.A., Chu Z.L., Ballard D.W. (1997). Phosphorylation of the PEST Domain of IκBβ Regulates the Function of NF-κB/IκBβ Complexes. J. Biol. Chem..

[44] McKinsey T.A., Brockman J.A., Scherer D.C., Al-Murrani S.W., Green P.L., Ballard D.W. (1996). Inactivation of IkappaBbeta by the Tax Protein of Human T-Cell Leukemia Virus Type 1: A Potential Mechanism for Constitutive Induction of NF-KappaB. Mol. Cell. Biol..

[45] DiDonato J., Mercurio F., Rosette C., Wu-Li J., Suyang H., Ghosh S., Karin M. (1996). Mapping of the Inducible IkappaB Phosphorylation Sites That Signal Its Ubiquitination and Degradation. Mol. Cell. Biol..

[46] Ghosh S., Baltimore D. (1990). Activation in Vitro of NF-κB” by Phosphorylation of Its Inhibitor IκB”. Nature.

[47] Chiao P.J., Miyamoto S., Verma I.M. (1994). Autoregulation of I Kappa B Alpha Activity. Proc. Natl. Acad. Sci. USA.

[48] Kearns J.D., Basak S., Werner S.L., Huang C.S., Hoffmann A. (2006). IκBε Provides Negative Feedback to Control NF-κB Oscillations, Signaling Dynamics, and Inflammatory Gene Expression. J. Cell Biol..

[49] Chen Y., Wu J., Ghosh G. (2003). KappaB-Ras Binds to the Unique Insert within the Ankyrin Repeat Domain of IkappaBbeta and Regulates Cytoplasmic Retention of IkappaBbeta x NF-KappaB Complexes. J. Biol. Chem..

[50] Totzke G., Essmann F., Pohlmann S., Lindenblatt C., Jänicke R.U., Schulze-Osthoff K. (2006). A Novel Member of the IκB Family, Human IκB-ζ, Inhibits Transactivation of P65 and Its DNA Binding. J. Biol. Chem..

[51] Sarais F., Rebl H., Verleih M., Ostermann S., Krasnov A., Köllner B., Goldammer T., Rebl A. (2020). Characterisation of the Teleostean κB-Ras Family: The Two Members NKIRAS1 and NKIRAS2 from Rainbow Trout Influence the Activity of NF-κB in Opposite Ways. Fish Shellfish Immunol..

[52] Andrea Oeckinghaus A., Post-ler T.S., Lienhard G.E. (2014). ΚB-Ras Proteins Regulate Both NF-κB-Dependent Inflammation and Ral-Dependent Proliferation. CellReports.

[53] Sangrador-Vegas A., Smith T.J., Cairns M.T. (2005). Cloning and Characterization of a Homologue of the Alpha Inhibitor of NF-κB in Rainbow Trout (*Oncorhynchus mykiss*). Vet. Immunol. Immunopathol..

[54] Geisler R., Bergmann A., Hiromi Y., Nüsslein-Volhard C. (1992). Cactus, a Gene Involved in Dorsoventral Pattern Formation of Drosophila, Is Related to the IκB Gene Family of Vertebrates. Cell.

[55] Lee Y., Umasuthan N., Whang I., Revathy K.S., Lee S., De Zoysa M., Oh C., Kang D.H., Noh J.K., Lee J. (2014). Two NF-κB Inhibitor-Alpha (IκBα) Genes from Rock Bream (*Oplegnathus fasciatus*): Molecular Characterization, Genomic Organization and MRNA Expression Analysis after Immune Stimulation. Fish Shellfish Immunol..

[56] Mai W., Huang F., Liu P. (2014). The Role of EaIκB-α, an IκB-α Homologue in Epinephelus Akaara, Involved in Innate Immune Response. Biotechnol. Lett..

[57] Gao R., Huang Y., Huang X., Guan L., Wei S., Zhou Y., Qin Q. (2014). Molecular Cloning and Characterization of Two Types of IκBα Orthologues in Orange-Spotted Grouper, Epinephelus Coioides. Fish Shellfish Immunol..

[58] Correa R.G., Tergaonkar V., Ng J.K., Dubova I., Izpisua-Belmonte J.C., Verma I.M. (2004). Characterization of NF-κΒ/IκΒ Proteins in Zebra Fish and Their Involvement in Notochord Development. Mol. Cell. Biol..

[59] Jakovlić I., Liu H., Wang W.-M. (2016). Identification, Origin and Evidence for Retained Functionality of Two IκBα Paralogs in Megalobrama Amblycephala. Dev. Comp. Immunol..

[60] Zhang M., Xiao Z., Sun L. (2012). Overexpression of NF-κB Inhibitor Alpha in Cynoglossus Semilaevis Impairs Pathogen-Induced Immune Response. Dev. Comp. Immunol..

[61] Wang Y., Wei H., Wang X., Du L., Zhang A., Zhou H. (2015). Cellular Activation, Expression Analysis and Functional Characterization of Grass Carp IκBα: Evidence for Its Involvement in Fish NF-κB Signaling Pathway. Fish Shellfish Immunol..

[62] Yazawa R., Kondo H., Hirono I., Aoki T. (2007). Cloning and Characterization of the IκBα Gene from Japanese Flounder, Paralichthys Olivaceus. Fish Shellfish Immunol..

[63] Feng J., Xu Y., Lin P., Peng X., Wang Y., Zhang Z. (2021). Identification of IκBα in Japanese Eel Anguilla Japonica That Impairs the IKKα-Dependent Activation of NF-ΚB, AP1, and Type I IFN Signaling Pathways. Dev. Comp. Immunol..

[64] Wang L., Zhou Z.-C., Guo C.-J., Rao X.-Y., Xiao J., Weng S.-P., Yin Z.-X., Yu X.-Q., He J.-G. (2009). The Alpha Inhibitor of NF-KappaB (IkappaBalpha) from the Mandarin Fish Binds with P65 NF-KappaB. Fish Shellfish Immunol..

[65] Singh P.P., Arora J., Isambert H. (2015). Identification of Ohnolog Genes Originating from Whole Genome Duplication in Early Vertebrates, Based on Synteny Comparison across Multiple Genomes. PLoS Comput. Biol..

[66] Morris R.T., Drouin G. (2011). Ectopic Gene Conversions in the Genome of Ten Hemiascomycete Yeast Species. Int. J. Evol. Biol..

[67] Campbell M.A., Hale M.C., McKinney G.J., Nichols K.M., Pearse D.E. (2019). Long-Term Conservation of Ohnologs Through Partial Tetrasomy Following Whole-Genome Duplication in Salmonidae. G3 Genes Genomes Genet..

[68] Reams A.B., Roth J.R. (2015). Mechanisms of Gene Duplication and Amplification. Cold Spring Harb. Perspect. Biol..

[69] Okamoto T., Ono T., Hori M., Yang J.P., Tetsuka T., Kawabe T., Sonta S. (1998). Assignment of the IkappaB-Beta Gene NFKBIB to Human Chromosome Band 19q13.1 by in Situ Hybridization. Cytogenet. Cell Genet..

[70] Eslamloo K., Xue X., Booman M., Smith N.C., Rise M.L. (2016). Transcriptome Profiling of the Antiviral Immune Response in Atlantic Cod Macrophages. Dev. Comp. Immunol..

[71] Sood N., Verma D.K., Paria A., Yadav S.C., Yadav M.K., Bedekar M.K., Kumar S., Swaminathan T.R., Mohan C.V., Rajendran K.V. (2021). Transcriptome Analysis of Liver Elucidates Key Immune-Related Pathways in Nile Tilapia Oreochromis Niloticus Following Infection with Tilapia Lake Virus. Fish Shellfish Immunol..

[72] Schiøtz B.L., Jørgensen S.M., Rexroad C., Gjøen T., Krasnov A. (2008). Transcriptomic Analysis of Responses to Infectious Salmon Anemia Virus Infection in Macrophage-like Cells. Virus Res..

[73] Zhang C., Jiang D., Wang J., Qi Q. (2021). The Effects of TPT and Dietary Quercetin on Growth, Hepatic Oxidative Damage and Apoptosis in Zebrafish. Ecotoxicol. Environ. Saf..

[74] Kaleo I.V., Gao Q., Liu B., Sun C., Zhou Q., Zhang H., Shan F., Xiong Z., Bo L., Song C. (2019). Effects of Moringa Oleifera Leaf Extract on Growth Performance, Physiological and Immune Response, and Related Immune Gene Expression of Macrobrachium Rosenbergii with Vibrio Anguillarum and Ammonia Stress. Fish Shellfish Immunol..

[75] Pacitti D., Lawan M.M., Feldmann J., Sweetman J., Wang T., Martin S.A.M., Secombes C.J. (2016). Impact of Selenium Supplementation on Fish Antiviral Responses: A Whole Transcriptomic Analysis in Rainbow Trout (*Oncorhynchus mykiss*) Fed Supranutritional Levels of Sel-Plex^®^. BMC Genom..

[76] Magray A.R., Ribera J.M., Isernhagen L., Galuska S.P., Günther J., Verleih M., Viergutz T., Brunner R.M., Ganai B.A., Ahmad F. (2022). Evaluation of Blood Cell Viability Rate, Gene Expression, and O-GlcNAcylation Profiles as Indicative Signatures for Fungal Stimulation of Salmonid Cell Models. Mol. Immunol..

[77] Lee S.H., Hannink M. (2002). Characterization of the Nuclear Import and Export Functions of IκBε. J. Biol. Chem..

[78] Zheng C., Yin Q., Wu H. (2011). Structural Studies of NF-ΚB Signaling. Cell Res..

[79] Jacobs M.D., Harrison S.C. (1998). Structure of an IkappaBalpha/NF-KappaB Complex. Cell.

[80] Sivasubramanian N., Adhikary G., Sil P.C., Sen S. (1996). Cardiac Myotrophin Exhibits Rel/NF-κB Interacting Activity in Vitro. J. Biol. Chem..

[81] Wolff B., Naumann M. (1999). INK4 Cell Cycle Inhibitors Direct Transcriptional Inactivation of NF-κB. Oncogene.

[82] Sarais F., Kummerow S., Montero R., Rebl H., Köllner B., Goldammer T., Collet B., Rebl A. (2021). PIAS Factors from Rainbow Trout Control NF-κB- and STAT-Dependent Gene Expression. Int. J. Mol. Sci..

[83] Rebl A., Rebl H., Verleih M., Haupt S., Köbis J.M., Goldammer T., Seyfert H.M. (2019). At Least Two Genes Encode Many Variants of Irak3 in Rainbow Trout, but Neither the Full-Length Factor Nor Its Variants Interfere Directly With the TLR-Mediated Stimulation of Inflammation. Front. Immunol..

[84] Chenna R., Sugawara H., Koike T., Lopez R., Gibson T.J., Higgins D.G., Thompson J.D. (2003). Multiple Sequence Alignment with the Clustal Series of Programs. Nucleic Acids Res..

[85] Huerta-Cepas J., Serra F., Bork P. (2016). ETE 3: Reconstruction, Analysis, and Visualization of Phylogenomic Data. Mol. Biol. Evol..

[86] Letunic I., Khedkar S., Bork P. (2021). SMART: Recent Updates, New Developments and Status in 2020. Nucleic Acids Res..

[87] Yang J., Zhang Y. (2015). Protein Structure and Function Prediction Using I-TASSER. Curr. Protoc. Bioinform..

[88] Pettersen E.F., Goddard T.D., Huang C.C., Meng E.C., Couch G.S., Croll T.I., Morris J.H., Ferrin T.E. (2021). UCSF ChimeraX: Structure Visualization for Researchers, Educators, and Developers. Protein Sci..

