# Supplementary material for: Lost and Found: The Family of NF-κB Inhibitors Is Larger than Assumed in Salmonid Fish
Source: Int J Mol Sci. 2023 Jun 16;24(12):10229. doi: 10.3390/ijms241210229 (PMC10299181; doi:10.3390/ijms241210229)
Supplement: Supplementary file 1 [file ijms-24-10229-s001.zip › ijms-2394373-supplementary.pdf]

Table S1. Sequence comparison across iκb proteins from rainbow trout *O. mykiss*.

Sequence 1	Sequence 2	Sequence Identity [%]
nfkbia-a1	nfkbia-a2	87.58
nfkbia-a1	nfkbia-b1	59.10
nfkbia-a1	nfkbia-b2	61.99
nfkbia-a1	nfkbia-c1	24.69
nfkbia-a1	nfkbia-c2.1	26.00
nfkbia-a1	nfkbia-c2.2	23.62
nfkbia-a2	nfkbia-b1	58.21
nfkbia-a2	nfkbia-b2	60.75
nfkbia-a2	nfkbia-c1	25.37
nfkbia-a2	nfkbia-c2.1	25.25
nfkbia-a2	nfkbia-c2.2	23.62
nfkbia-b1	nfkbia-b2	85.37
nfkbia-b1	nfkbia-c1	25.55
nfkbia-b1	nfkbia-c2.1	27.59
nfkbia-b1	nfkbia-c2.2	24.50
nfkbia-b2	nfkbia-c1	25.74
nfkbia-b2	nfkbia-c2.1	26.04
nfkbia-b2	nfkbia-c2.2	23.46
nfkbia-c1	nfkbia-c2.1	85.89
nfkbia-c1	nfkbia-c2.2	82.10
nfkbia-c2.1	nfkbia-c2.2	92.42
nfkbie-a1	nfkbie-a2	81.64
nfkbid-a1	nfkbi-da2	82.04
nfkbiz-a1.1	nfkbiz-a1.3	86.33
nfkbiz-a1.1	nfkbiz-a1.4	86.33
nfkbiz-a1.1	nfkbiz-a2.1	99.44
nfkbiz-a1.3	nfkbiz-a1.4	100.00
nfkbiz-a1.3	nfkbiz-a2.1	85.77
nfkbiz-a1.4	nfkbiz-a2.1	85.77
nfkbia-a1	nfkbie-a1	27.68
nfkbia-b1	nfkbie-a1	29.58
nfkbia-c1	nfkbie-a1	29.27
nfkbia-c2.1	nfkbie-a1	30.73
nfkbia-a1	nfkbie-a2	29.78
nfkbia-b1	nfkbie-a2	29.13
nfkbia-c1	nfkbie-a2	28.12
nfkbia-c2.1	nfkbie-a2	29.83
bcl3-a1	bcl3-a2	85.28

Table S2. Location of different primers for qPCR analysis

**Gene (ltranscript isoforms)**	**Location of primers for qPCR analysis (forward primer + reverse primer)**
nfkbia-a (nfkbia-a1 + nfkbia-a2)	NM_001124368 942 GCCTGTCTGGTGATGAACAGATGTACGATGACATTACATTTGGTCAGAATTGAAGCAGTCGCATCACGTGGTGGTGTGAAGTCAAGTCAATGAA<br>XM_021600117 981 GCATGTCTGATGATGAACAGATGTACGATGACATTACATTCGGGCAGAATTGAAGTGGTCGCATCACATGGTGATGTGAAGTCAAGTCGATGAA<br>NM_001124368 GCTGCTACTGGCCTGTCCAGTCCAATCAAAGCACAGTGGGAACTCCAGGTCCCAGAAGCC 1094<br>XM_021600117 GCTGCTACGGGCCAGTCCAGTC-----AAAGCACAGTGGGAACTCCAGGTCCCAGAAGCC 1129
nfkbia-b (nfkbia-b1 + nfkbia-b2)	XM_021574049 640 ACCCAGCTCCCAGCCATCATGGCCACACCAAACTACAGTGGTCAGAACTACAGTGGTCAGAACTGTTTGCATCTGGTCTCTATCCATGGCTTTC<br>XM_021585138 604 ACCCAGCTCCCAGCCATTATGGCCACACCAAACTACAGTGGTCAGAACTACAGTGGTCAGAACTGTTTGCATCTGGTCTCTATCCATGGCTTCC<br>XM_021574049 TCTCGCTTGTGGAGAGCCTTGTTGCTCTCGGAGCTGACATCGATGCACAGGAGCAG 774<br>XM_021585138 TCTCGCTAGTGGAGAGGCTTGTTGATCTCGGAGCTGACATCGATGCACAGGAGCAG 738
nfkbia-c (nfkbia-c1 + nfkbia-c2.1 + nfkbia-c2.2)	XM_021623282 1156 GGGAGCTGAGGCAGGACTGTGTACGAGAGATCACCTCCAACTGCCAGAGCACTGACTACCTGCATGTCACCAACTACTCAGGGGTGAGTGC 1246<br>XM_021623283 1156 GGGAGCTGAGGCAGGACTGTGTACGAGAGATCACCTCCAACTGCCAGAGCACTGACTACCTGCATGTCACCAACTACTCAGGGGTGAGTGC 1246<br>XM_021618304 1137 GGGAGCTGAGGCAGGACTGTGTACGAGAGATCACCTCCAACTGCCAGAACACCGACTATCTATACGTCACCAACTACTCGGGGGTGAGTGC 1227
nfkbid-a1	XM_036970146 992 GTATGAGAGATGGGGTCCTATGGATGAGCACCAAGCCCTCTTGGCCATACAGGAAGGTCCATTCTCCCCTATGGGGAACAATTACTTCT<br>XM_036970146 CCAGCCCATCCTCCTCTTTGGACTACAGCCATACTCCGGCCTACAGCCCTCAGATGGTCTCCAGCTATAACACACAGCAGATGCA 1165
nfkbid-a2	XM_021625427 1654 AGGTTGAATCCAGACATCTGTACTTCCAAGAGGGAGGGATTCTAGCGTATCTTCATGTGAGGATTTTCAAACACGGCAAATCTCTAATATATA<br>XM_021625427 CTAGGGCATTCACTTTGAAACTATATGTTTGATTGACATGATTTTGGTCACTATCTGAATTCAGTTCTGAATTTAATAATGGCTAGCTAGTAA<br>XM_021625427 TGAGC 1844
nfkbie-a1	XM_021600115 1607 CTGTAGGGTTATTTATCGTTGTTGTGTGGTGTGTGTGTGTATATGTGAGTGTGTGGACTATTTAGATACTCACATTGAAAAGTTAATTCT<br>XM_021600115 CTGCTAGCAAAGTGGTAC 1714
nfkbie-a2	XM_021611602 290 GCAACCGCTACCTTTGGTTTCAGAAAGGAACAGTGAATTCCTACTCAGGTGTGATCGAGTTTTCCTCCCGA<br>XM_021611602 GAACTGCTCGCACCATCAAGCAACATATAGACCTCTCGGGGATTAAACCGGTCAAGACTACCTGGAGTG 429
nfkbiz-a1.1 + nfkbiz-a2.1	XM_036951335 1478 TCGTCAATGTCAAGGCATTCAGTGGAAACACAGCACTGCACTTTGCCAGTTCTGTGCATGGTCGCCTGACTCAGGTAGATGCTGTG<br>XM_036982243 1476 TCGTCAATGTCAAGGCATTCAGTGGAAACACAGCACTGCACTTTGCCAGTTCTGTGCATGGTCGCCTGACTCAGGTAGATGCTGTG<br>XM_036951335 AAACTGCTGATGAGGAGAGGGGCCGACCCCAGCTCCAAGAACCTGGAGAATGAGCAGC 1621<br>XM_036982243 AAACTGCTGATGAGGAGAGGGGCTGACCCCAGCTCCAAGAACCTGGAGAATGAGCAGC 1619
nfkbiz-a1.3 + nfkbiz-a1.4	XM_036982244 1834 TTCTGAGCTGACAAACAGTGTTCCCATTGTTCATTGTTTTTTGGTCATCAGGAGGTAAGTTAACTAAACCCTCTAACATGAGTTC 1918<br>XM_036982245 1803 TTCTGAGCTGACAAACAGTGTTCCCATTGTTCATTGTTTTTTGGTCATCAGGAGGTAAGTTAACTAAACCCTCTAACATGAGTTC 1887
bcl3-a	XM_021591521 3032 GCCAGTCGTACAGTGGGAACACGGCCCTGCACAGTGCCTGTGGGCGAGGCCAGGTAGACACAGCCAGGCTGCTACTGAAGAA<br>XM_021624309 1766 GCCAGTCGTACAGTGGGAACACGGCCCTGCACAGTGCCTGTGGGCGAGGCCAGGTAGACACAGCCAGGCTGCTACTGAAGAA<br>XM_021591521 CAGAGCCGACAGCAGTGTGAAGAACTACCACAATGACACCCCCGCGATGGTGGCCAAGAACAAGAAGGTAACAGATGT 3191<br>XM_021624309 CAGAGCCGACAGCAGTGTGAAGAACTACCACAATGACACCCCTGCGATGGTGGCCAAGAACAAGATGGTAACAGATGT 1925

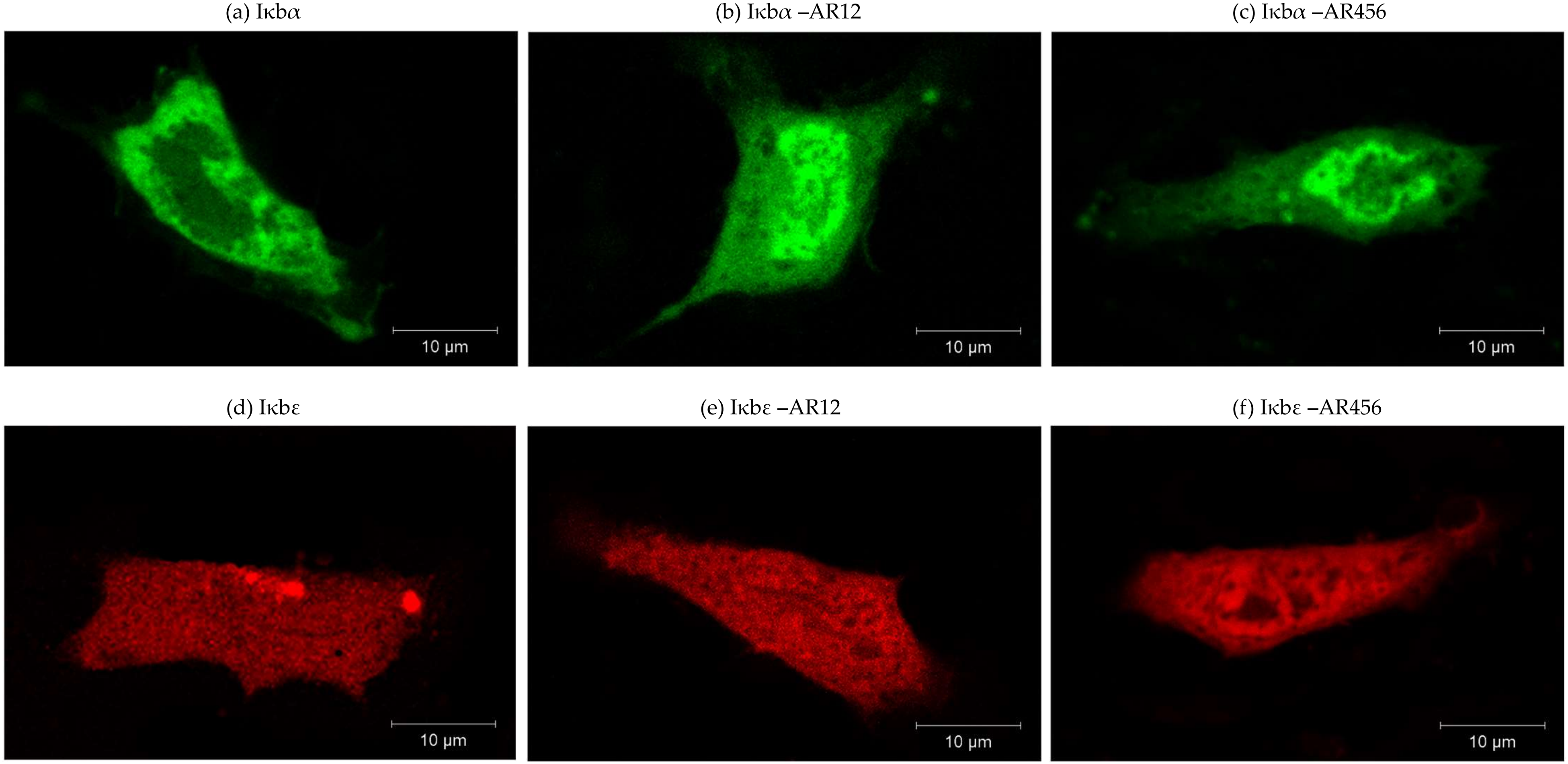

Figure S1. Overexpression of gfp-tagged iκbα (green fluorescence) or plum-tagged iκbε constructs (red fluorescence) in salmonid model cells. Confocal analysis of (a) iκbα, (b) ankyrin repeats 1 and 2 of iκbα, (c) ankyrin repeats 4, 5 and 6 of iκbα, (d) iκbε (red), (e) ankyrin repeats 1 and 2 of iκbε, (f) ankyrin repeats 4, 5 and 6 of iκbε in CHSE-214. White scale bar represents 10 μm.
